# Assets and challenges facing caregivers when managing malaria in young children in rural Uganda

**DOI:** 10.1186/s12936-016-1521-1

**Published:** 2016-09-13

**Authors:** Rosemin Kassam, John Collins, Richard Sekiwunga

**Affiliations:** 1 Faculty of Medicine, School of Population and Public Health, University of British Columbia, 2206 East Mall, Vancouver, BC V6T 1Z3 Canada; 2 Department of Educational Studies, Faculty of Education, University of British Columbia, 2125 Main Mall, Vancouver, V6T 1Z4 Canada; 3 Child Health and Development Centre, School of Medicine, Makerere University, P.O. Box 7062, Kampala, Uganda

**Keywords:** Malaria, Treatment-seeking, Behaviour, Child, Scale, Caregiver, Assets, Challenges, Uganda

## Abstract

**Background:**

Despite efforts to improve malaria management for children, a substantial gap remains between policy and practice in Uganda. The aim of this study was to create quantitative profiles of assets and challenges facing caregivers in Butaleja District when managing malaria in children aged 5 years and under. The objectives were: (1) to estimate caregivers’ assets and challenges during an acute episode; and, (2) to ascertain which caregiver attributes influenced receipt of an appropriate anti-malarial the most.

**Methods:**

Data from a 2011 cross-sectional, household survey and ten psychometrically justified scales were used to estimate caregivers’ assets and challenges. The scales scores were simple counts across a series of items, for example, the number of times a caregiver answered a knowledge item correctly or the number of times a caregiver relied on a credible source for information. Since high scores on six of the scales reflected attributes that eased the burden of caregiving, these were labelled ‘caregiver assets’. Similarly, high scores on four of the measures signalled that a caregiver was having trouble managing the malaria episode, thereby reflecting deficits, and these were labelled ‘caregiver challenges’. ANOVAs were used to compare scale scores between caregivers of children who received an appropriate anti-malarial versus those who did not.

**Results:**

On the six asset scales, caregivers averaged highest on knowledge (65 %), followed by correct episode management (48 %), use of trustworthy information sources (40 %), ability to initiate or redirect their child’s treatment (37 %), and lowest on possible encounters with health professionals to assist in treatment decisions (33 %). Similarly, the average caregiver reported problems with 74 % of the issues they might encounter in accessing advice, and 56 % of the problems in obtaining the best anti-malarial. Caregivers whose children received an appropriate anti-malarial demonstrated greater assets and fewer challenges than those whose child did not, with important regional differences existing. Overall, no one region performed particularly well across all ten scales.

**Conclusions:**

Findings from this study suggest that the low use of artemisinin-based combination therapy (ACT) in Butaleja for children 5 years and under may result from caregivers’ high perceived barrier to accessing ACT and low perceived benefits from ACT.

**Electronic supplementary material:**

The online version of this article (doi:10.1186/s12936-016-1521-1) contains supplementary material, which is available to authorized users.

## Background

Malaria remains the leading cause of morbidity and mortality in Uganda, resulting in 25–40 % of annual outpatient visits and nearly half of inpatient childhood deaths [1]. Recent health system data and studies conducted in various regions of Uganda support either a rising trend or one that has remained steady among children aged 5 years and under [2]. Given the pivotal role case management continues to play in the management of malaria, the Uganda government has over the years introduced a number of national policies and programmes with the aim, by 2010/2015, to have 85 % of children under five with suspected or confirmed malaria receive anti-malarial treatment within 24 h of initial symptoms [1, 3]. Examples of such initiatives include the dissemination of first-line anti-malarial artemisinin-based combination therapy (ACT) cost-free from all public health facilities; the introduction of the Integrated Community Case Management Programme (iCCM) in mid-2010 to bring diagnostics and treatment closer to the community; the introduction of the Affordable Medicines Facility-Malaria (AMFm) in Spring 2011 to improve access to a range of ACT from licensed public and private outlets; the ban of resistant anti-malarials; the training of public providers; sensitization meetings with district-level leaders; and information, education and communication campaigns to improve household awareness [1, 4]. Despite these initiatives, translating policy into practice to ensure universal access to ACT has been challenging in rural areas due to regular stock-outs of anti-malarials at public facilities, dispensing of non-first-line anti-malarial therapy at health facilities, and limited access to licensed private outlets supported by the AMFm scheme [5–7]. At the time of this study, it was also recognized that the delivery of iCCM was complex, requiring both providers and recipients of care to be fully engaged for desired outcomes to be achieved [6, 8].

Research in Uganda has supported the notion that inadequate caregiver treatment-seeking practices in the context of a weak health system are important elements limiting the use of ACT in children 5 years and under [9]. In spite of knowing this, most interventions have been aimed at improving the formal health system. The few that have focused on households have been aimed principally at increasing awareness through mass campaigns and sensitization meetings. Although these initiatives have generated some improvement in the usage of ACT for young children, they have not produced sufficient behaviour change to achieve the 2010/2015 national target of 85 % [10]. One reason for their limited impact may be the simplistic view that changing awareness will automatically change behaviour [11, 12]. While awareness about malaria and its treatment is necessary to making an informed decision, the idea of awareness directly influencing behaviour does not consider many other factors that might influence treatment-seeking behaviour [13]. Ecological frameworks propose that a multiplicity of factors co-exist at the individual, family, community, and societal levels, interacting to influence behaviour. Critics of these earlier efforts advocate a multi-level, system-strengthening approach that considers all factors likely to influence uptake of new services and programmes by caregivers [6, 12].

Central to most public health programmes is to start with the health issue affecting a target population and to explore what factors might be perpetuating this issue. The intent is to understand how and to what extent these factors might be influencing the problem, to select factors which, if modified, will result in alleviating the health issue, to implement programme(s) and intervention(s) that can mitigate these factors, and to evaluate the impact of the intervention(s) at alleviating the health concern [13]. Accordingly, the current study responds to community-based desires to improve malaria care for the very young in the rural Butaleja District of Uganda.

As a first step, a household survey was conducted across Butaleja District in 2011 to describe caregivers’ treatment-seeking behaviour for malaria in children 5 years and under and to estimate the use of ACT and of appropriate anti-malarials in this age group. Analysis of the findings established a substantial gap between the national malaria policy and practices in Butaleja, with only 41 % of children with fever and presumed malaria reported to have received an ACT, 21 % received any blood test whatsoever, and 32 % received an appropriate anti-malarial [14]. Next, guided by the health belief model, a parallel study was carried out to operationalize caregivers’ assets and challenges with the management of malaria in young children and to conduct psychometrically analysis of the scales (Kassam et al., pers. comm.) [15]. The health belief model is a value-expectancy theory which posits that health behaviours are adopted based on assessments about benefits and barriers to action articulated as six key elements: (1) perceived susceptibility to the illness; (2) perceived severity of the illness; (3) perceived benefits of the therapeutic intervention; (4) perceived barriers; (5) cues to action; and, (6) self-efficacy (the belief that one can successfully execute the action) [15]. The aim of the current study was to use the psychometrically justified scales to create quantitative profiles of assets and challenges facing caregivers in Butaleja District when managing malaria in children 5 years and under. Specifically, the objectives were: (1) to estimate caregivers’ assets and challenges during an acute episode; and, (2) to ascertain which caregiver attributes influenced receipt of an appropriate anti-malarial the most. These objectives were assessed across the district and within different regions of the district.

## Methods

### Study design

Data from a cross-sectional household survey conducted in 2011 and ten psychometrically justified scales were used to estimate assets and challenges facing caregivers when managing malaria in children 5 years and under in Butaleja District, Uganda [14]. Ethics approval for the project and written informed consent from participants was obtained prior to conducting the surveys.

### Setting

Butaleja District is located in rural eastern Uganda. At the time of this study, the district consisted of ten sub-counties (mostly rural) and two town councils (peri-urban) [16]. The district’s population for 2010 was estimated at 206,300, with about 41,240 households and 44,300 children aged under 5 years [16]. The sub-county of Mazimasa has the largest population, whereas Himutu and Butaleja sub-counties have the lowest populations [16]. The Banyole tribe represented the major ethnic group at 66 %, followed by the Bagwere tribe at 5 % and the Jopadhola tribe at 3 %, with other (Basoga, Bagisu, Iteso, Baganda, Banyankole, Acholi) tribes comprising less than 2 % [16]. The three predominant religious groups include Protestants (53 %), Muslims (30 %), and Catholics (17 %) (K Mweru, pers. comm.). Lunyole is the predominant spoken language. A large majority of the population practices subsistence farming [17]. While almost 40 % of the population lives below the poverty line, poverty is a society-wide phenomenon demonstrated through indicators, such as insecurity, low quality public services, scarcity of jobs, and lack of physical, technical and health information throughout society [17]. The average literacy rate among persons above 10 years is estimated to be 63 %, with males more literate (71.6 %) than females (54.0 %) [16].

The district normally experiences two major rainfall periods between the months of May and October, although in the recent past it has suffered irregular and unpredictable rainfall patterns that resulted in severe flooding of much of the district, creating swamps, submerging gardens, destroying roads, and leaving many families homeless [17]. With the emergence of trading centres and towns being set-up across the district without adequate physical planning for health and environmental implications, most of the district lacks adequate solid waste disposal systems and latrines, most roadside eating houses operate under unhygienic conditions, and school sanitation is below the expected standard [17]. The weather, together with its geographical terrain and unplanned growth, makes Butaleja District a hyper-endemic malaria area with high risk of year-round malaria transmission.

The public health infrastructure in Uganda is stratified into four levels by district, sub-county, parish, and village [18]. At the highest level there is one public hospital: for Butaleja this is located in Busolwe town council. At the next lower level are Health Centre IIIs, regulated to provide a range of inpatient and outpatient care and outreach services, and Health Centre IIs which provide limited outpatient care. At the lowest level are Health Centres I, which exist as informal structures consisting of volunteers elected by villagers [known as village health teams or community health workers (CHWs)] to provide basic health services at the community level. While the national policy professed access to free medicine from all levels of the health system, including ACT supplied as Coartem^®^, at the time of this study ACT had not yet been disseminated to CHWs (K Mweru, pers. comm). Other artemisinin-based combinations could also be purchased from private outlets. The district, however, has no pharmacies. Thus, anti-malarials are sold largely by the few licensed drug shops located mostly in town centres and market areas and by unlicensed private outlets located in villages across the district that do not have formal training in the management of malaria.

### Study population and sampling

A purposive, multi-step sampling process was used to ensure representation across all ten sub-counties and two town councils [14]. The sampling process considered size of villages, religious denominations, dominant tribes, and proximity to a government health centre. At village level, households were recruited using a simple random process to avoid self-selection. Household surveys were carried out with 424 caregivers who met the following inclusion criteria: they had at least one child 5 years or younger who had been febrile within the previous 2 weeks; they were the primary care provider for the child (which included supervision, bathing and feeding); they resided within Butaleja District; they spoke the common district dialect (Lunyole); they agreed to participate; and they willingly signed the consent form using thumbprint or written signature. Survey questions about current practice were asked with reference to the youngest child with fever (referred to as the ‘index child’). All households were excluded if their children’s fevers were confirmed by a qualified health professional to be associated with an illness other than malaria.

### Data collection

This study used response data from household surveys conducted in 2011. Guided by the six asset and challenge elements of the health belief model, the literature on caregiver treatment-seeking behaviour, measurement experts, and existing survey instruments, seven educational and environmental factors identified a priori were incorporated in the survey. These factors included malaria-related knowledge (disease and treatment), episode management, assistance with critical decision, access to information sources, problems with accessing advice, problems with obtaining the best anti-malarial, and perceived ability to initiate/redirect actions. The final printed survey encompassed interviewer instructions, research assistants’ post-interview remarks, and 160 questions collecting information on the seven educational and environmental factors, household identification, child’s disease presentation, demographics/socio-economic status. The development of the survey instrument, administration, and quality control measures are described in detail elsewhere [14].

This study aimed to develop quantitative profiles of assets and challenges facing caregivers when managing malaria in children 5 years and under during an acute episode. The intent of the profiles was to yield insight to broader answers of educational and environmental importance beyond caregivers’ responses to individual survey items and beyond simple yes/no or present/absent dichotomies. For example, if caregiver knowledge was a factor, then to use a psychometrically validated scale for knowledge to determine on average how well caregivers in the district perform on this factor, and which caregivers possessed relatively more knowledge and which relatively less?

To this end, ten psychometrically justified scales developed in a parallel study were used to create these profiles (Kassam et al., pers. comm). Six of the scales were labelled ‘caregiver assets’, as high scores on these scales reflected attributes which eased the burden of caregiving: (1) precursors to receiving an appropriate anti-malarial (α = .68); (2) episode management (α = .74); (3) caregiver knowledge (α = .51); (4) assistance with critical decisions from health professionals (α = .65); (5) reliable information sources (α = .64); and, (6) initiating or redirecting treatment (α = .65). The remaining four scales were labelled ‘caregiver challenges’, as high scores on these scales signaled that the caregiver was having trouble managing the malaria episode, thereby reflecting deficits: (1) lack of information sources (α = .59); (2) lack of assistance with critical decisions (α = .41); (3) problems accessing advice (α = .75); and, (4) problems obtaining best anti-malarial (α = .68). Eight of the ten scales’ reliabilities exceed Nunnally’s α = .60 rule-of-thumb of acceptable internal consistency for newly developed measures [19, 20].

### Measures and analysis

#### Quantitative profiles of caregivers’ assets and challenges

Survey data were transcribed from completed survey documents onto Excel spreadsheets for cleaning and verification, and subsequently transported into SPSS^®^ v21 files for analysis. The scales scores are simple counts across a series of items. For example, for caregiver knowledge scale—the number of times a caregiver answered a knowledge item correctly, for reliable information sources scale—the number of times they relied on a credible source for information, for problems accessing advice scale—the number of times they reported problems issues they might have encountered when accessing advice, or for lack of assistance with critical decisions scale—the numbers of times they lacked any support or assistance and were forced to rely on “no one else (myself)”. Since raw scale scores are strongly affected by the numbers of items in each scale (7–19), re-scaled scores are reported throughout. These re-scaled scores are calculated by dividing the raw scores by the number of possible correct responses in each scale, therefore, representing the percentage of affirmatively scored items in each scale. One-way ANOVAs were used to compare overall scale scores between caregivers of children who received an appropriate anti-malarial versus those who did not for all ten scales.

#### Sensitivity analysis

The sensitivity analysis (Table 1) assessed whether the scales and/or the demographic characteristics: (1) discriminated between caregivers of children who received an appropriate anti-malarial versus those who did not, and (2) distinguished among different regions (sub-counties and town councils). Successive one-way ANOVAs followed by η^2^ tested each of the scales, whereas χ^2^ followed by η^2^ tested select demographic variables, using receipt of appropriate anti-malarial and sub-county as the independent variables.

**Table 1:** Sensitivity of scales, SES and demographics in predicting receipt of an appropriate anti-malarial and regional differences

Benchmark → (Scale) Dependent Variables↓	Receipt of an appropriate anti-malarial^a^				Sub-county/town council location			
	df	F	p value		df	F	p value	
*Behaviour scales: assets*								
Precursors to receiving an appropriate anti-malarial	1419	112.03	<.000	.211	11,410	4.60	<.000	.110
Episode management	1419	264.93	<.000	.387	11,410	6.66	<.000	.152
Caregiver knowledge	1419	25.45	<.000	.057	11,410	2.18	.015	.055
Assistance with critical decisions from health professional	1419	40.92	<.000	.089	11,410	5.34	<.000	.124
Reliable information sources	1419	1.56	.213	.004	11,410	2.52	.004	.063
Initiating/redirecting actions	1419	.90	.764	.000	11,410	1.81	.051	.046
*Behaviour scales: challenges*								
Lack of reliable information sources (relying on self)	1419	.12	.726	.000	11,410	2.56	.004	.064
Lack of assistance with critical decisions	1419	42.20	<.000	.093	11,410	2.40	.020	.053
Problems accessing advice	1415	.21	.645	.001	11,406	1.47	.140	.038
Problems obtaining best anti-malarial	1416	15.34	<.000	.036	11,407	1.59	.098	.041
*Household wealth (SES)*								
Dwelling permanence	1419	.12	.726	.000	11,410	3.13	<.000	.077
Rurality	1419	.39	.532	.001	11,410	8.37	<.000	.183

Caregiver demographics	df	χ^2^	p value		df	χ^2^	p value	
Religion	3	4.41	.222	.000	33	138.20	<.000	.073
Tribe	7	6.43	.494	.000	77	182.22	<.000	.023
Gender	1	4.12	.042	.010	11	13.25	.277	.032
Age category	6	1.73	.943	.003	66	74.30	.226	.024
Education	4	5.35	.253	.000	44	70.14	.007	.047
*Benchmark criteria*								
Receipt of an appropriate anti-malarial^a^	–	–	–	–	12	24.12	.012	.057
Sub-county	12	24.12	.012	.015	–	–	–	–

^a^Appropriate anti-malarial is defined as having received only age-specific first-line malaria treatment

Appropriate anti-malarial is defined as having received only Uganda’s age-specific, first-line treatment for uncomplicated and severe malaria [14]. For children under 4 months old, this included quinine (oral or injectable) or artesunate (injectable or rectal) therapy. For children 4 months and older, this included receiving any ACT and/or artesunate (injectable or rectal) or quinine (injectable) [21]. For each child, an audit of all medications reported to have been given by the caregiver to manage the current febrile illness determined whether the appropriate anti-malarial criteria had been met.

Demographic measures included gender, age, education, occupation, religion, tribe, household size, number of children 5 years old and under, caregiver’s relationship to head of household and to index child, current employment, and socio-economic status (SES). Following Filmer et al. [22, 23], SES was inferred using 16 measures of housing, water, sanitation, energy sources, transportation equipment, communication devices, information sources, and livestock counts. The SPSS v21 categorical principal component analysis (CATPCA) was used to examine the 16 household attributes. Two large, uncorrelated principal components reflecting household wealth index emerged: (1) dwelling permanence, ranging from less permanent to more permanent household building features (18.9 % principal component variance); and, (2) durable assets (11.6 % principal component variance), reflecting a geographic location axis from remote or isolated households to those residing in relatively more urban settings (rurality).

## Results

### Characteristics of the sample

A total of 424 caregivers across Butaleja District participated in the interview-assisted survey. The mean (SD) age of caregivers was 31 ± 10 years. A large majority of caregivers were female (86.0 %) and mother to the index child (79.0 %). While caregivers were diverse with respect to tribe and religion, the majority belonged to the Banyole tribe (74.5 %), and the three main religious groups represented were protestants (49.8 %), Muslim (32 %) and Catholics (13.4 %). Most caregivers reported having a low educational level, with 83.3 % completing primary level or less of schooling. Over 90 % of caregivers reported earning no regular wages (79.6 % of males and 95.8 % of females), and the large majority of caregivers (73.5 % female and 11.2 % male) reported farming as their primary occupation. Almost half of the households occupied mud-structured houses or homes with only one room, a large majority used traditional pit latrines located outside the home, approximately three-fifths reported owning a radio, and about one-third reported not having any form of transportation. The mean (SD) age of the index child was 22 ± 16 months, most represented the youngest child in the household, and the proportion of boys to girls was equal. A large majority (82.6 %) of caregivers expressed at the time of the survey that they suspected their child had malaria at the outset of symptoms.

### Quantitative profiles of caregivers’ assets and challenges

**Fig. 1:**
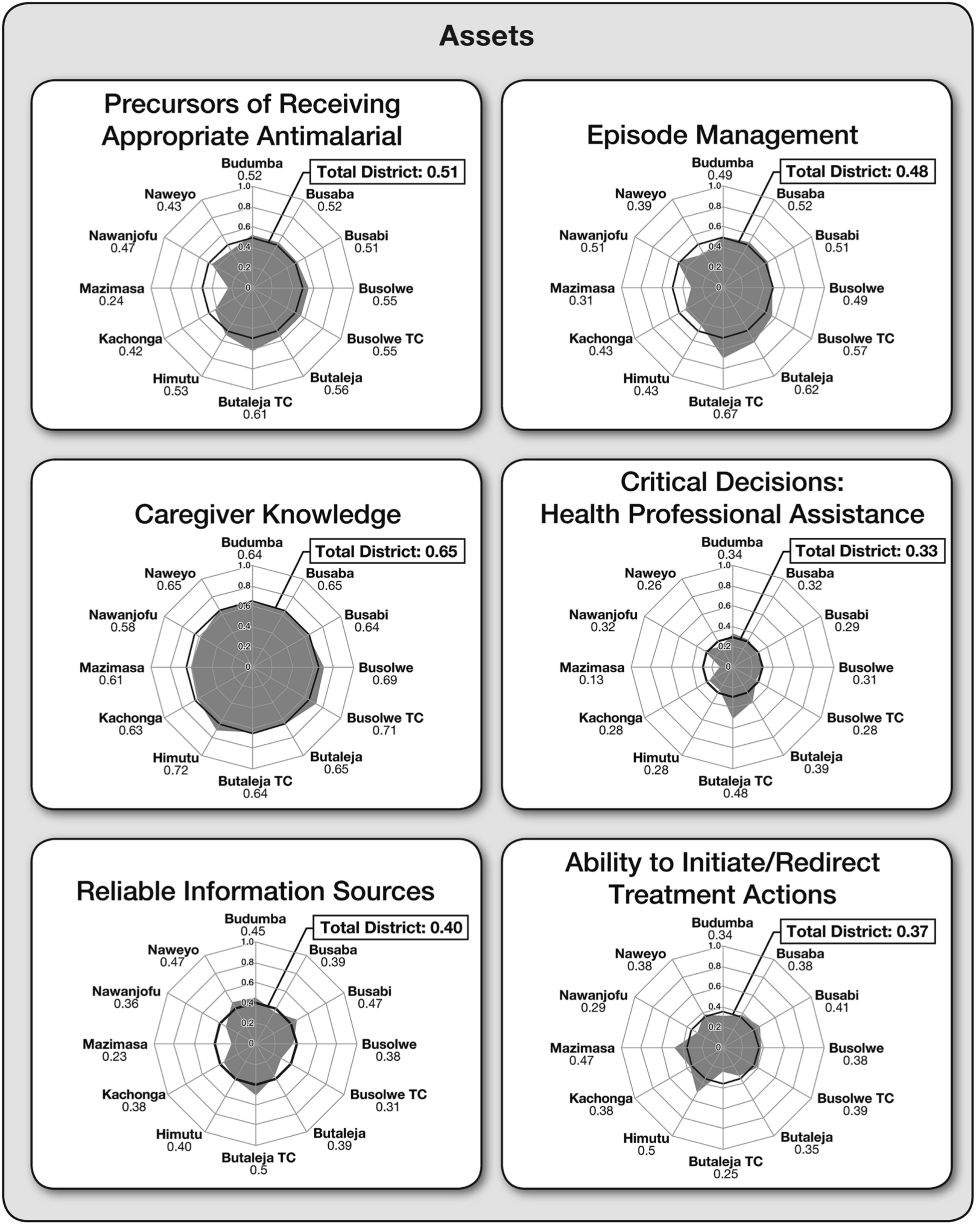
Six asset scales: total scores and scores across different regions of Butaleja District

**Fig. 2:**
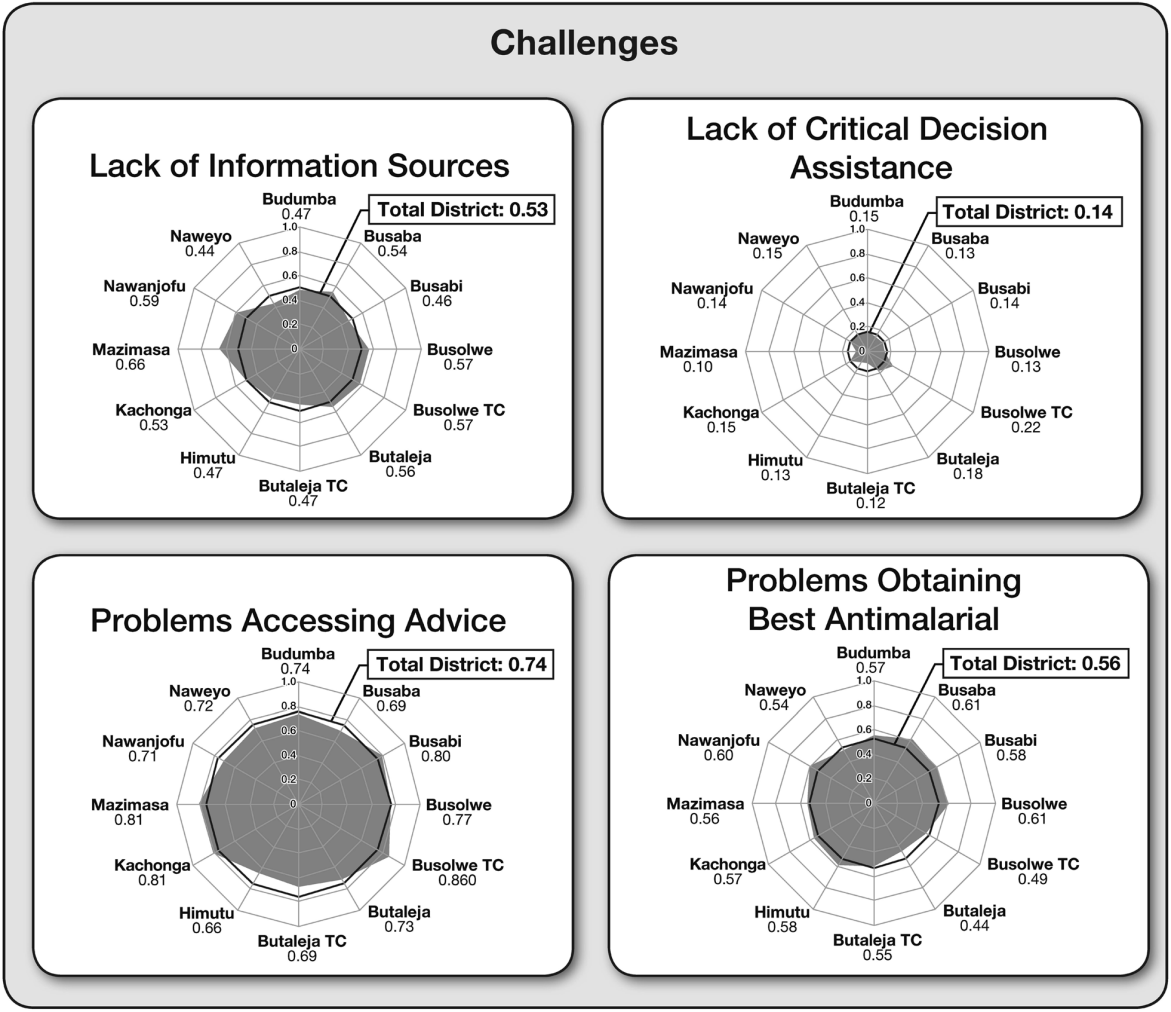
Four challenge scales: total scores and scores across different regions of Butaleja District

For the asset scales, only rarely did a caregiver correctly identify all the items comprising any single scale. Of the 424 caregivers, only four correctly identified all 17 caregiver knowledge items; two had accessed credible sources on all eight information items, and ten claimed the ability to initiate or redirect their child’s treatment on all seven scale items. The average caregiver performance was also considerably low (Additional file 1). Of the six asset scales, caregivers scored highest on the caregiver knowledge scale, where on average they responded correctly to about 65 % of the items, followed by 48 % correct for episode management scale. Additionally, caregivers on average accessed trustworthy information sources for 40 % of the eight possible instances, they were able to initiate or redirect treatment for 37 % of the seven designated intervention opportunities, but they only received help from trained health professionals with 33 % of the 11 possible intervention opportunities. Similarly, the average caregiver reported problems with 74 % of the seven issues they might encounter in accessing advice about treatment for their child, and 56 % of the nine problems areas with obtaining the best anti-malarial. Caregivers reported that they themselves were their sole information source for 53 % of eight different information-gathering encounters and 14 % of nine critical assistance-seeking occasions. As expected, caregivers with higher asset scores obtained overall lower challenge results (r = −.250; p < .000). Figures 1 and 2 illustrate the six asset and four challenge profiles of Butaleja caregivers as radar charts, respectively, contrasting where the gaps in treatment-seeking behaviours are most acute. Overall, results showed that the average caregiver accumulated less than half the total possible number of asset points (45.1 %) and about half the possible number of challenge points (49.2 %), although there was considerable spread within both assets (SD = 13.4 %) and challenges (SD = 11.0 %).

### Ten scales and appropriate anti-malarial prediction

Of the 424 caregivers, 290 reported that their child had received an anti-malarial (68.4 %), but closer inspection showed that only 134 children received an appropriate anti-malarial (31.6 %). Four of the six caregiver asset scales predicted significantly whether a child actually received an appropriate anti-malarial (Table 1), and two of the four caregiver challenge scales predicted significantly whether a child received an appropriate anti-malarial. Table 1 confirms that receipt of an appropriate anti-malarial is better predicted by four of the six asset scales and two of the four challenge scales than by any of the demographic characteristics, except for caregiver gender. Thus, in health belief model terms, perceptions and preparedness outweighed demographics as triggers to seeking an appropriate anti-malarial.

**Fig. 3:**
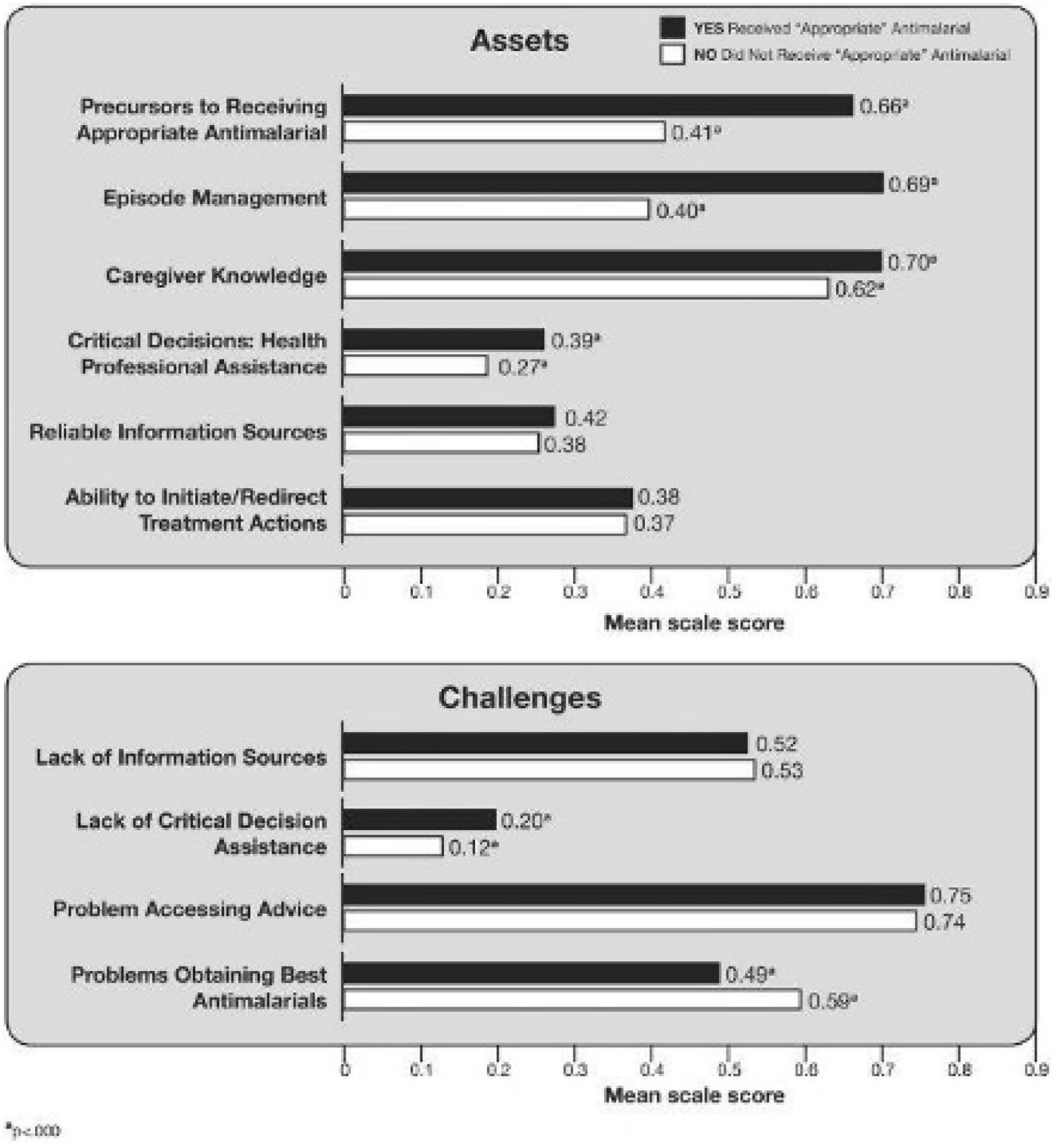
Asset and challenge scores: caregivers whose child did and did not receive an appropriate anti-malarial

Figure 3 highlights asset and challenge scale differences for caregivers whose child did and did not receive an appropriate anti-malarial. On the four caregiver asset scales that significantly predicted receipt of an appropriate anti-malarial, the average caregivers whose child did receive an appropriate anti-malarial were 25 % higher on the precursors to receiving an appropriate anti-malarial scale than those whose child did not (66.0 vs 41.5 %). Caregivers of children who received an appropriate anti-malarial performed better on three additional asset scales: (1) they scored 29 % higher on the episode management scale (68.7 vs 40.1 %); (2) they demonstrated 8 % higher knowledge scores (69.9 vs 62.2 %); and, (3) they reported 12 % more assistance from health professionals at moments of critical decision-making (38.6 vs 27.0 %).

There were also differences for two of the four challenge categories they faced. Caregivers whose child did not receive an appropriate anti-malarial reported 11 % higher scores on the problems obtaining the best anti-malarial than recipients of an appropriate anti-malarial (59.4 vs 48.8 %). An unexpected pattern observed in this study was that caregivers of children who received an appropriate anti-malarial reported a 7 % greater tendency to rely only on themselves (not health professionals nor family nor neighbours), than caregivers whose child did not receive an appropriate anti-malarial (19.7 vs 12.3 %), thus lacking critical assistance when making decisions during an acute episode.

### Ten scales and sub-county and town council (regional) differences

Seven of the ten scales (five of the six asset scales and two of the four challenge scales) were found to be significant discriminators among sub-counties and town councils, highlighting differences in preparedness, resources and challenges within these communities for managing malaria in young children when the illness strikes over and over again (Table 1). Accordingly, the ten scales tell a somewhat different story across the sub-counties than simple rates of appropriate anti-malarial administration. The ten scales highlighting regional differences in assets and challenges influencing caregivers’ treatment-seeking behaviour serve as a useful guide for where public health interventions ought to begin. For example, public education programmes need to begin in locations where management practices are poor, precursors of receiving an appropriate anti-malarial are weak, caregiver knowledge is under-realized, critical decision assistance is not readily available, or where information is in short supply.

**Fig. 4:**
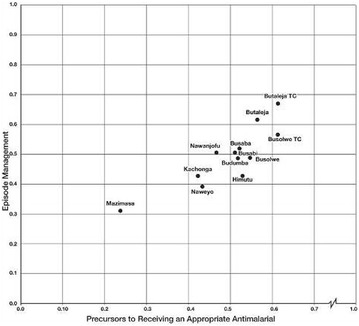
Arraying precursors to receiving an appropriate anti-malarial with episode management scale scores across Butaleja District

In general, Figs. 1, 2 and 4 are helpful in clarifying where in the district the needs are greatest and public health interventions are most pressing.

Overall, no one region performed particularly well across all ten scales. Figures 1 and 2 summarize these results as ‘radar plots’. The plots allow for a visual comparison between sub-counties, highlighting where in the district the needs are greatest and public health interventions most pressing. Larger shaded areas represent higher assets or challenges for each sub-county (spokes). Thus, the large shaded areas for the knowledge or precursors scales contrast sharply with few reliable information sources or restricted professional assistance with critical. Lopsided or asymmetrical plots indicate high scores in shaded areas and low scores for unshaded sub-counties.

Commonly, the town councils and their surrounds reported favourably (comparatively high assets and comparatively low challenges). Notably, caregivers residing in Butaleja (both town council and sub-county) scored higher on episode management and on precursors to receiving an appropriate anti-malarial scales, and they made greater use of professional assistance than did caregivers in any other location. Of all the different regions, Mazimasa sub-county fared worse across five of the six asset scales and highest on the challenge scale of lack of information sources.

Figure 4 highlights where public education needs are greatest by arraying sub-counties with precursors to receiving an appropriate anti-malarial scale scores, and sub-counties with where caregivers reside and by episode management scale scores. Four sub-counties where precursors are not in place and where management practices are weakest are those locations needing immediate public interventions to equip caregivers with ‘how-to’ practices required for prompt action (Mazimasa, Kachonga, Naweyo, Himutu). The distribution of assets and challenges across the regions would indicate Mazimasa to be in greatest need of public health interventions, and it might be one of the locations to begin such interventions.

## Discussion

This study is the first to examine caregivers’ assets and challenges to inform where public health interventions need to begin to improve the management of malaria in Uganda for children 5 years and under. Overall, the study established that: (1) the average caregiver performance was considerably low; (2) caregivers whose children received an appropriate anti-malarial demonstrated greater assets and fewer challenges than those whose child did not; and, (3) important regional differences existed within the district for asset and challenge factors influencing treatment-seeking behaviour. While the health belief model highlights the interplay between perceived susceptibility, barriers and benefits with the modifying factors of cues to action and demographic variables, this study found that the strongest links existed between treatment-seeking aspects of ‘knowing what to do and why’ and obtaining an appropriate anti-malarial for the child. For the most part, demographics such as caregiver gender, age, schooling, religion, tribe, or household wealth (in terms of either dwelling permanence or asset type) made little difference.

### Episode management of a current febrile illness

As evidenced by their episode management scale score average of 48 %, caregivers in Butaleja exhibited a mediocre level of managerial proficiency. While most caregivers introduced some form of action within 24 h of noticing first symptoms, for three-quarters of the children this constituted home management, and a quarter relying on traditional herbs [14]. The high reliance on home management in Butaleja compared to elsewhere in Uganda is particularly concerning, given that home management, and in particular the use of traditional herbs, has been associated with delayed care-seeking at health facilities and with CHWs [18, 24, 25]. Evaluation of caregivers’ management practices determined that a large majority of caregivers needed to continue treatment with subsequent action, suggesting most children did not improve with home management alone.

Additionally, despite the higher rate of care seeking at public facilities reported in this study (50 %) compared to what has been reported for other districts in eastern Uganda (17–26 %) or nationally (21–24 %), four out of seven caregivers obtained their anti-malarial medicines from private outlets (primarily unlicensed vendors) [18, 26–29]. These findings confirm reports that private outlets continue to fill the gap between home management, CHWs and public facilities. Regular ACT stock-outs, frequent referrals to private vendors by public health providers to purchase anti-malarials, and poor service at public health facilities have been cited as common reasons for visiting private outlets in Butaleja [7]. Further, only a third of caregivers in Butaleja reported that ACT was easy to find in their community and one in six believed ACT was affordable.

### Knowledge about malaria and ACT

Assessment of caregivers’ knowledge provided additional valuable insight into caregivers’ perceived benefits and further explanation for the low ACT usage across Butaleja. While average caregiver knowledge was the highest of any scale (65 %), there was considerable variability and substantial knowledge deficiencies in certain key topic areas. In health belief model terms, the results demonstrate that Butaleja caregivers acknowledged high susceptibility for malaria, since about four out of five caregivers readily attributed their index child’s fever to malaria. However, evaluation of the caregiver knowledge scale indicated that caregivers across Butaleja perceived the benefits of ACT to be low, with just one in three caregivers reporting they would select an ACT if given the choice. The low preference for ACT was likely contingent on their low awareness about ACT. Only one in three caregivers was aware that ACT was the government-recommended, first-line anti-malarial or that it cured malaria the best, and only one in four was able to name a setting where they were certain to get ACT for free if they required it. The proportion of caregivers who reported knowing that ACT was the first-line anti-malarial was substantially lower in Butaleja than had been previously reported in a national study (34 vs 57 %), although a comparable proportion reported ACT as the anti-malarial which cures malaria the best [29].

### Sources of information

Most national programmes for educating the public on malaria and its treatment in Uganda rely on primary sources such as: news media (newspapers, television, radio), sensitization programmes using trained health providers, and informed community members such as chairpersons [1, 30, 31]. Results from the study’s two information source scales show that, across Butaleja, such national programmes have had negligible influence on caregivers’ treatment-seeking behaviours. Caregivers could name a primary source for only about a third of the eight topic areas pertinent to malaria and its treatment. Hearsay from community members, families and/or themselves was the common resource when a primary source was not mentioned. Collective findings from the two information source scales and the knowledge scale suggest that information delivered by primary sources either fall short of conveying key messages or that information is not transmitted in a fashion where key messages are easily accessible, retrievable or understood by caregivers.

### Challenges to treatment-seeking

The high scores observed on two of the challenge scales may provide one explanation for caregivers’ preference for home management in Butaleja [14]. Given the high level of perceived obstacles to seeking external advice and treatment, it is not surprising that a large majority of caregivers used home management which is convenient, and traditional herbs which can be obtained cost-free from neighbouring fields. For seven of the nine items, half of caregivers expressed challenges with obtaining the best anti-malarial, with caregivers whose child did not receive an appropriate anti-malarial reporting a higher scale score than recipients of an appropriate anti-malarial. On the other hand, the comparatively high score on the problems with accessing advice scale suggests that caregivers across Butaleja perceived barriers related to accessing advice to be substantial, irrespective of whether their child received an appropriate anti-malarial. Collectively, across the two challenge scales, common obstacles included: getting permission to seek advice or treatment, finding the time, knowing where to access advice, best anti-malarial, and health providers, lack of money, finding transportation, availability of medicines, and distance to health facilities.

While few studies in Uganda have mentioned time and permission as a challenge to seeking advice or treatment, several Ugandan and sub-Saharan African studies have reported access to money, transportation and distance to be important barriers to treatment seeking for caregivers of children under 5 years [9, 18, 27, 32–34]. A possible reason for the lack of time and need for permission may be explained by women caregivers’ multiple responsibilities in rural settings. In Butaleja, most caregivers are peasant farmers who also manage domestic chores and are responsible for childcare, leaving them with little time to seek prompt advice or treatment outside the home. Consequently, seeking external care to manage a malaria episode often has numerous ramifications, some of which include loss of productivity from farming, neglect of usual household tasks, and re-allocation of assets to pay for care [35]. Obstacles to seeking advice and treatment can be particularly taxing when a child falls ill during the farming season, heavy rains when roads become difficult to travel, and if they have to secure transportation and money for medicines [36]. Such circumstances require additional time and effort, as well as implicit or explicit permission from their spouse who usually has control over household resources and finances [9, 28, 36, 37].

### Assistance with decision-making

Caregivers’ decision-making was further compromised by low levels of interpersonal cues available to them during periods of acute illness when critical decisions were made. The low 33 % score (range: 0–76 %) for the assistance with critical decisions from health professionals onals scale suggests that trained health providers’ influence was either limited or not sought, despite three out of four caregivers indicating it was their preference to first seek advice from a health provider. Health providers were most likely to be consulted regarding selection of an anti-malarial, followed by advice about when to start an anti-malarial. However, well over half of all initial care decisions were made by caregivers on their own or with help from family members. Children whose caregivers received assistance from trained health professionals were 12 % more likely to receive an appropriate anti-malarial, than those who did not. Unfortunately, in this study as in others, seeking care from trained health providers mainly occurred after failure with initial care [9, 28]. Given that health provider assistance with critical decision resulted in increased receipt of appropriate anti-malarial, future public health interventions need to promote early contact with trained health providers who have access to ACT.

Although seemingly counter-intuitive, caregivers whose children received an appropriate anti-malarial reported 7 % more instances of having only themselves to rely on when making critical decisions than those whose child did not. One explanation for such an association between reliance-on-self and receipt of an appropriate anti-malarial may be that some of those caregivers knew they lacked resources, thus were more determined and successful at obtaining an appropriate anti-malarial. Further investigation is needed to confirm this relationship, especially in remote areas where health professional services are infrequent.

### Public health interventions required to improved health delivery

The average precursors to receiving an appropriate anti-malarial scale score of 51 % revealed a moderately low utilization of opportunities and local resources characteristic of a child receiving an appropriate anti-malarial [14]. Given that malaria is a recurrent infection, with children under 5 years experiencing an average of four episodes per year, in households averaging 2.3–2.8 children the concept of self-efficacy is central and exerts a strong influence on whether a caregiver is able to carry out a desirable behaviour with each new infection [38–40]. Future educational and other public health strategies aimed at improving management of malaria in young children should, therefore, involve both caregivers and their family members. Shaping caregivers’ and their family members’ knowledge, expectations and personal capacities to promote use of appropriate anti-malarials will also influence and improve health providers’ (including vendor) practices. In circumstances where health providers are prescribing based on presumptive diagnoses or are dispensing ineffective anti-malarials, it is likely that caregivers who have a preference for ACT and understand the importance of having a confirmed malaria diagnosis will be more likely to search out and request such treatment [41, 42]. On the other hand, those who lack the information to make informed management decisions may limit the demand for effective interventions even when such interventions are available. However, the literature on health literacy suggests that emphasis on factual information alone may not be sufficient to change health behaviours, such information needs to be disseminated in a manner that is locally relevant, acceptable and engaging to change caregivers’ behaviour [43, 44]. Future public health programmes will need to simultaneously enhance caregivers’ personal abilities to make appropriate decisions by enabling them to understand, judge, sift, and use factual information in the context of their own circumstances.

### Limitations

The findings from this study need to be considered in the context of potential limitations. While a rigorous methodology was used to minimize biases, such as recall, misclassification and social desirability biases, such biases are inherent to all surveys that involve behaviour research. In this study, where researchers relied on caregivers’ self-reported information, recall bias was minimized by focusing on the index child’s last fever episode in the previous 2 weeks of the survey. Misclassification of medicines was minimized by confirming what caregivers reported against photographs printed on laminated posters of medicines used commonly for malaria in Butaleja, and social desirability bias was controlled by assuring caregivers that their information would remain confidential and by ensuring privacy during the interview.

While the quantitative profiles developed in this study provide a unique perspective on caregivers’ assets and challenges when managing acute malaria episodes, the reliability of the lack of assistance with critical decisions scale was below criterion, suggesting less than optimal internal consistency and/or homogeneity. Though Cronbach’s alpha values below .5 are generally considered poor, it is also acknowledged that the desired degree of scale reliability needs to be considered within the context of the research, whether the research is exploratory versus applied, and if the results are intended to make low versus high stake decisions [20]. It is also established that a low alpha does not negate results obtained from that scale. Accordingly, for this exploratory study, where the primary intent was to develop quantitative profiles to assess caregivers’ assets and challenges with managing malaria, it can be persuasively argued that all scales were worthy of examination. All caregivers need to be equipped with adequate illness management practice skills, correct knowledge, access to information sources, and appropriate cues to action, in order to manage future episodes of fever in the index child or with other children. With this in mind, the lack of assistance with critical decisions scale was retained for analysis as it provided valuable insight on the extent to which critical decisions were being made in isolation by caregivers during periods of acute episodes of malaria. Nevertheless, despite their contribution, further research involving the ten scales is encouraged to improve their reliability and generalizability.

## Conclusions

Using the health belief model as a template to evaluate caregivers’ treatment-seeking behaviour provides helpful guidance for intervention strategies. The health belief model is a value-expectancy theory, which states that individuals are most likely to adopt a desired action if they see that assessed benefits outweigh assessed barriers. The results from this study suggest that Butaleja’s caregivers’ seemingly low motivation to change treatment-seeking behaviours may result from high perceived barriers to, and low perceived benefits from ACT. Thus, two sets of interventions are required: one to minimize challenges in obtaining advice and treatment, and the other to improve caregivers’ perceived benefits about ACT and their ability to navigate the health system to obtain ACT in a prompt and efficient fashion.

## Additional file

10.1186/s12936-016-1521-1 Additional Tables 2.1–2.7.

## Abbreviations

ACT: artemisinin-based combination therapy
AMFm: Affordable Medicines Facility-Malaria
iCCM: Integrated Community Case Management
CATPCA: Categorical Principal Component Analysis
CHW: Community Health Workers
ANOVA: analysis of variance
χ2: chi square test
η2: eta-squared
SES: socio-economic status
