# Supplementary material for: Assets and challenges facing caregivers when managing malaria in young children in rural Uganda
Source: Malar J. 2016 Sep 13;15(1):467. doi: 10.1186/s12936-016-1521-1 (PMC5020540; doi:10.1186/s12936-016-1521-1)
Supplement: Supplementary file 1 — 10.1186/s12936-016-1521-1 Additional Tables 2.1–2.7. [file 12936_2016_1521_MOESM1_ESM.docx]

**Additional file 5**

**Table 2.1.** Asset scale: Precursors to receiving an appropriate anti-malarial (α=.68)

| **Item-by-Item Scale Summary^a^** | **Response Categories^a^** | **Item/Scale Score (n=424)** | |
| --- | --- | --- | --- |
|  |  | **Mean** | **SD** |
| Awareness that government policy recommends ACT | ACT | 0.34 | 0.48 |
| Which AMs cure the best | ACT | 0.35 | 0.48 |
| Given the choice, which AM would you select first | ACT | 0.32 | 0.47 |
| Was child seen by a HP | Yes | 0.70 | 0.46 |
| Where was child seen by HP | PHF | 0.50 | 0.50 |
| Was medicine used for subsequent action | Yes | 0.84 | 0.37 |
| Where was AM (normally) obtained | Regulated outlets^b^ | 0.53 | 0.50 |
| Were western medicines kept in home over last 6 months for future use | Yes | 0.56 | 0.50 |
| Types of medicines kept as home remedy for future use on day of the survey | ACT | 0.43 | 0.50 |
| **Raw Score** |  | 4.57 | 2.26 |
| **Re-Scaled Score (of possible 9 points)** |  | **0.51** | **0.25** |

^a^Abbreviations: anti-malarial (AM); artemisinin combination therapy (ACT); public health facility (PHF); trained health professional (HP).

^b^Regulated outlets: community health worker; PHF; regulated private outlets.

^c^Number of respondents reflects those to whom question was asked based on conditional branching.

**Table 2.2** Asset scale: Caregiver knowledge (α=.51)

| **Item-by-Item Scale Summary^a^** | **Response**  **Categories^ab^** | **Item/Scale Score (n=424)** | |
| --- | --- | --- | --- |
|  |  | **Mean** | **SD** |
| **Knowledge About Malaria** | | | |
| What are the main symptoms of malaria | Classical (fever/chills)  Other symptoms  Wrong answer | 1.05 | 0.90 |
| What is the one main cause | Mosquitoes  Mosquito surrogates  Wrong answer | 1.59 | 0.79 |
| **Knowledge About AMs** | | | |
| How soon after fever best to start AM | Within 24hrs | 0.95 | 0.21 |
| Which AM cures best | ACT | 0.35 | 0.48 |
| Given choice, which AM select first | ACT | 0.32 | 0.47 |
| Which AM cures the worst | Non-ACT | 0.95 | 0.22 |
| Given choice, which AM select last | Non-ACT | 0.92 | 0.27 |
| When is it best to stop AM | Adverse effect/as directed | 0.32 | 0.49 |
| **Knowledge About National Policies** | | | |
| Awareness gov’t policy recommends | ACT | 0.34 | 0.48 |
| How soon after onset of fever to start AM | Within 24hrs | 0.71 | 0.46 |
| Is starting AM within 24-hours of fever realistic | Yes | 0.93 | 0.25 |
| **Where Can AMs and Advice be Accessed** | | | |
| Which outlets have the best quality medicines | Regulated outlets^c^ | 0.75 | 0.43 |
| Where are ACTs available for free | PHF/CHW | 0.75 | 0.43 |
| Can you get ACT for free when you need it | Free | 0.33 | 0.47 |
| Best individuals to obtain advice from on malaria | HP/CHW | 0.78 | 0.41 |
| **Raw Score** |  | 10.99 | 2.58 |
| **Re-Scaled Score**  **(of possible 17 points)** |  | **0.65** | **0.15** |

^a^Abbreviations: anti-malarial (AM); artemisinin combination therapy (ACT); community health worker (CHW); government (gov’t); public health facility (PHF); trained health professional (HP).

^b^Calculating item Mean Score: items with 3 response categories result in 3 point scale: 2 points; 1 points; 0 points.

^c^Regulated outlets: CHW; PHF; regulated private outlets.

**Table 2.3** Asset scale: Episode management (α=.74)

| **Item-by-Item Scale Summary^a^** | **Response**  **Categories^ab^** | **Item/Scale Score (n=424)** | |
| --- | --- | --- | --- |
|  |  | **Mean** | **SD** |
| **First Action Care** | | | |
| When was first action started | 6 hrs  6-24hrs  >24hrs | 1.02 | 0.51 |
| Ever received first line or second line AM (usage) | First line AM  Second line AM  Other | 0.44 | 0.73 |
| **Subsequent Action Care** | | | |
| Was medicine used for subsequent action | Yes | 0.84 | 0.37 |
| Ever received first line or second line AM (usage) | First line AM  Second line AM  Other | 0.84 | 0.92 |
| **Care Over the Course of the Illness** | | | |
| Was child seen by a HP | Yes | 0.70 | 0.46 |
| Where was the child seen by HP | PHF | 0.50 | 0.50 |
| Was blood test done | Yes | 0.21 | 0.41 |
| Ever received first line or second line AM (usage) | First line AM  Second line AM  Other | 1.00 | 0.92 |
| One main reason for choosing this AM (referred to what child was given) | Rational reasoning^c^ | 0.52 | 0.50 |
| How long after 1^st^ symptom was AM started | Within 24 hrs | 0.52 | 0.50 |
| Where was AM (normally) obtained | Regulated Outlets^d^ | 0.53 | 0.50 |
| **General Practice** | | | |
| Are ACTs easy to find | Yes | 0.33 | 0.47 |
| Are ACTs affordable | Yes | 0.17 | 0.37 |
| Were AM kept in home over last 6 months for future use | Yes | 0.56 | 0.50 |
| ACT in the home on the day of the survey | Yes | 0.43 | 0.50 |
| Given choice, where would obtain AM from | Regulated outlets^d^ | 0.94 | 0.24 |
| **Raw Score** |  | 9.34 | 4.06 |
| **Re-Scaled Score (of possible 20 points)** |  | **0.48** | **0.21** |

^a^Abbreviations: anti-malarial (AM); artemisinin combination therapy (ACT); community health worker (CHW); public health facility (PHF); trained health professional (HP).

^b^Calculating item Mean Score: items with 3 response categories resulting in 3 point scale: 2 points; 1 points; 0 points.

^c^Rational reasoning: most effective; prescribed by trained HP; always 1^st^ choice; cures malaria; saves life; to treat malaria; recommended on radio.

^d^Regulated outlets: CHW; PHF; regulated private outlets.

^e^Number of respondents reflects those to whom question was asked based on conditional branching.

**Table 2.4** Assistance with critical decisions from health professionals (α=.65) vs. self-dependence (α=.41)

|  | **Asset Scale:**  **Assistance with Critical Decisions from**  **Health Professionals** | | | **Challenge Scale:**  **Lack of Assistance with**  **Critical Decisions (Self)** | | |
| --- | --- | --- | --- | --- | --- | --- |
| **Item-by-Item Scale Summary^a^** | **Response Categories^a^** | **Item/Scale Score (n=424)** | | **Response Categories^a^** | **Item/Scale Score (n=424)** | |
|  |  | **Mean** | **SD** |  | **Mean** | **SD** |
| **Assistance with Index Child’s Illness** | | | | | | |
| Which individual assisted you with recognizing child was sick | HP | 0.02 | 0.15 | Self | 0.3 | 0.4 |
| Which individual assisted you with recognizing child suffered from malaria | HP | 0.26 | 0.43 | Self | 0.2 | 0.4 |
| **Assistance with Initiating Care in Index Child** | | | | | | |
| Who advised you on starting the traditional medicine | HP | 0.00 | 0.04 | Self | 0.1 | 0.3 |
| Who advised you on starting any home remedy | HP | 0.06 | 0.24 | Self | 0.4 | 0.5 |
| Who advised you on starting medicine from external source | HP | 0.06 | 0.24 | Self | 0.3 | 0.5 |
| **Assistance with Selecting AM Regimen** | | | | | | |
| Who decides what form of action to start | HP | 0.24 | 0.43 | -- | -- | -- |
| Who decided to give this AM | HP | 0.65 | 0.78 | Self | 0.1 | 0.3 |
| Who decides which AM to start | HP | 0.68 | 0.47 | -- | -- | -- |
| Who decides when AM can be started | HP | 0.48 | 0.56 | -- | -- | -- |
| Who decided when to stop this AM | HP | 0.40 | 0.70 | Self | 0.4 | 0.6 |
| Given a choice, who would you go to first for advice | HP | 0.78 | 0.41 | Self | 0.0 | 0.1 |
| Where would money come from | -- | -- | -- | Self | 0.4 | 0.5 |
| **Raw Score** |  | 3.63 | 1.99 |  | 1.90 | 1.47 |
| **Re-Scaled Score**  **(of possible points)** |  | **0.33**  **(of 11 points)** | **0.18** |  | **0.14**  **(of 9 points)** | **0.11** |

^a^Abbreviations: anti-malarial (AM); trained health professional (HP).

^b^Number of respondents reflects those to whom question was asked based on conditional branching. Home remedy includes home-stock medicines and supportive care.

**Table 2.5** Information sources scales: accessing reliable sources (α=.64) vs. self-dependence (α=.59)

|  | **Asset Scale:**  **Reliable Information Sources** | | | **Challenges Scale:**  **Lack of Information Sources (Self)** | | |
| --- | --- | --- | --- | --- | --- | --- |
| **Item-by-Item Scale Summary^a^** | **Response Categories^b^** | **Item/Scale Score (n=424)** | | **Response Categories** | **Item/Scale Score (n=424)** | |
| ***Where did you learn the following …*** | | **Mean** | **SD** |  | **Mean** | **SD** |
| **Information About Malaria** |  |  |  |  |  |  |
| About malaria | Primary source  Secondary source  Hearsay | 1.09 | 0.99 | Self | 0.30 | 0.46 |
| **Information About AMs** | | | | | | |
| This AM was the best  (Caregiver’s selected best) | Primary source  Secondary source  Hearsay | 0.76 | 0.96 | Self | 0.52 | 0.50 |
| This AM was the worst  (Caregiver’s selected worst) | Primary source  Secondary source  Hearsay | 0.25 | 0.66 | Self | 0.81 | 0.39 |
| **Information About National Policy** | | | | | | |
| What AM does the government recommend | Primary source  Secondary source  Hearsay | 0.61 | 0.92 | Self | 0.68 | 0.47 |
| When it is best to start an AM | Primary source  Secondary source  Hearsay | 0.91 | 0.99 | Self | 0.44 | 0.50 |
| What does the government recommend regarding when best to start an AM | Primary source  Secondary source  Hearsay | 1.10 | 0.99 | Self | 0.41 | 0.49 |
| Where ACT can be obtained | Primary source  Secondary source  Hearsay | 1.19 | 0.98 | Self | 0.31 | 0.46 |
| About good and poor quality AM medicines | Primary source  Secondary source  Hearsay | 0.41 | 0.81 | Self | 0.75 | 0.43 |
| **Raw Score** |  | 6.32 | 3.93 |  | 4.22 | 1.89 |
| **Re-Scaled Score**  **(of possible points)** |  | **0.40**  **(of 16 points)** | **0.26** |  | **0.53**  **(of 8 points)** | **0.24** |

^a^Abbreviation: anti-malarial (AM); artemisinin combination therapy (ACT).

^b^Calculating item Mean Score: items with 3 response categories resulting in 3 point scale: 2 points; 1 points; 0 points.

**Table 2.6** Challenge scales: Problems accessing advice (α=.75) and obtaining the best anti-malarial (α = .68)

|  | **Challenge Scale:**  **Problems Accessing Advice** | | | **Challenge Scale:**  **Problems obtaining Best Antimalarial** | |
| --- | --- | --- | --- | --- | --- |
| **Item-by-Item Scale Summary** | **Response Categories** | **Scale Score (n=424)** | | **Scale Score (n=424)** | |
|  |  | **Mean** | **SD** | **Mean** | **SD** |
| Knowing where to go | Problem | 0.81 | 0.39 | 0.44 | 0.50 |
| Getting permission to go | Problem | 0.93 | 0.26 | 0.43 | 0.50 |
| Finding transportation | Problem | 0.59 | 0.49 | 0.58 | 0.49 |
| Access to health professional | Problem | 0.68 | 0.47 | 0.54 | 0.50 |
| Access to female health professional | Problem | 0.74 | 0.43 | 0.50 | 0.50 |
| Finding time | Problem | 0.84 | 0.36 | 0.50 | 0.50 |
| Resources within the community | Problem | 0.61 | 0.49 | -- | -- |
| Availability of medicine | Problem | -- | -- | 0.69 | 0.45 |
| Health facility is too far | Problem | -- | -- | 0.65 | 0.48 |
| Getting money for medicine | Problem | -- | -- | 0.70 | 0.46 |
| **Raw Score** |  | 5.21 | 1.86 | 5.04 | 2.34 |
| **Re-Scaled Score**  **(of possible points)** |  | **0.74**  **(of 7 points)** | **0.27** | **0.56**  **(of 9 points)** | **0.26** |

**Table 2.7.** Asset scale: Ability to initiate/redirect child’s treatment actions (α=.65)

| **Item-by-Item Scale Summary^a^** | **Response Categories** | **Scale Score (n=424)** | |
| --- | --- | --- | --- |
|  |  | **Mean** | **SD** |
| To start independently on a different form of first action | Yes | 0.63 | 0.48 |
| Who makes decision on what form of action to take | Self | 0.27 | 0.45 |
| To independently start on a different western AM | Yes | 0.33 | 0.47 |
| Who makes final decision which western AM to start | Self | 0.06 | 0.25 |
| To independently decide when to start a western AM | Yes | 0.63 | 0.48 |
| Who makes final decision when AM can be started | Self | 0.31 | 0.46 |
| Can you independently decide to stop AM | Yes | 0.38 | 0.48 |
| **Raw Score** |  | 2.61 | 1.79 |
| **Re-Scaled Score**  **(of possible 7 points)** |  | **0.37** | **0.26** |

^a^Abbreviation: anti-malarial (AM).
